# Anti-aging and tyrosinase inhibition effects of *Cassia fistula* flower butanolic extract

**DOI:** 10.1186/s12906-016-1484-3

**Published:** 2016-12-03

**Authors:** Pornngarm Limtrakul, Supachai Yodkeeree, Pilaiporn Thippraphan, Wanisa Punfa, Jatupol Srisomboon

**Affiliations:** 1 Department of Biochemistry, Faculty of Medicine, Chiang Mai University, Chiang Mai, 50200 Thailand; 2 Department of Obstetrics & Gynecology, Faculty of Medicine, Chiang Mai University, Chiang Mai, 50200 Thailand

**Keywords:** Collagenase, Collagen synthesis, Glycosaminoglycan, Hyaluronic acid, Matrix metalloproteinase

## Abstract

**Background:**

Natural products made from plant sources have been used in a variety of cosmetic applications as a source of nutrition and as a whitening agent. The flowers of *Cassia fistula* L, family Fabaceae, have been used as a traditional medicine for skin diseases and wound healing and have been reported to possess anti-oxidant properties. The anti-aging effect of *C. fist*ula flower extract on human skin fibroblast was investigated.

**Methods:**

The butanolic extraction of *C. fistula* flowers was completed and the active compounds were classified. The cytotoxicity of fibroblasts was evaluated by SRB assay for the purposes of selecting non-toxic doses for further experiments. The collagen and hyaluronic acid (HA) synthesis was then measured using the collagen kit and ELISA, respectively. Moreover, the enzyme activity, including collagenase, matrixmelloproteinase-2 (MMP-2) and tyrosinase, were also evaluated.

**Results:**

It was found that the flower extract did not affect skin fibroblast cell growth (IC_50_ > 200 μg/mL). The results did show that the flower extract significantly increased collagen and HA synthesis in a dose dependent manner. The flower extract (50–200 μg/mL) also significantly inhibited collagenase and MMP-2 activity. Furthermore, this flower extract could inhibit the tyrosinase activity that causes hyperpigmentation, which induces skin aging.

**Conclusions:**

The *C. fistula* flower extract displayed a preventive effect when used for anti-aging purposes in human skin fibroblasts and may be an appropriate choice for cosmetic products that aim to provide whitening effects, and which are designated as anti-aging facial skin care products.

## Background

Skin aging is a complicated biochemical progression resulting from many individual intrinsic and extrinsic factors such as age, hormones and exposure to UV light [1]. The progression occurs in the epidermal and dermal layers and is mainly related to extracellular matrix (ECM) degradation [2]. The enzymes involved in ECM degradation are matrix metalloproteinases (MMPs) such as gelatinases (MMP-2) and collagenase [3]. Skin loses its tensile strength due to the effect of ECM degradation by MMPs. In this process, the wrinkling of skin occurs and roughness and dryness also markedly arise along with certain pigment abnormalities such as hypo- or hyper-pigmentation [2, 4]. For the treatment of hyper-pigmentation, tyrosinase inhibitors have been investigated. Tyrosinase is a rate-limiting enzyme that converts tyrosine to melanin [5]. Tyrosine inhibitors thus play an importance role as skin-lightening agents [6] while hyaluronic acid (HA) synthesis regulates skin hydration and the occurrence of wrinkles. HA, a non-sulphated glycosaminoglycan (GAG), is composed of repeating units of disaccharides including D-glucuronic acid and N-acetyl-D-glucosamine [7]. HA also regulates the repair of tissues, including the enhancement of the immune system response by activation of inflammatory cells and in response to the injury of fibroblasts [8–10].

Natural products made from plant sources such as *Glycyrrhiza glabra* [11] or green tea [12] have been used in cosmetic applications as a source of nutrition and as a whitening agent. A significant amount of evidence has pointed to the beneficial effects of orally or topically administered phytochemicals from plant extracts, especially with regard to the improvement of skin conditions. Some examples of the beneficial effects are that skin aging and skin inflammation can be reduced. *C. fistula* (golden shower), family Fabaceae, is found in numerous Asian countries such as Thailand, China, Myanmar and India. In previous studies, *C. fistula* flower extract was found to possess antioxidant, anticancer, antibacterial, antifungal, and antidiabetic properties [13, 14]. The effect of *C. fistula* in Ayurvedic medicine is known to be involved with treating various disorders including, skin diseases, leprosy, haematemesis, pruritus and diabetes [15, 16]. Various parts of *C. fistula* have displayed pharmacological properties [17]. The flower, seed, fruit and pulp have been used to treat skin diseases including leprosy [18]. The pulp has been recognized for its antidiabetic properties [15] and has been used in a tonic that has been applied in treatments of gout and rheumatism [19]. The leaves and ripe pods have been traditionally used as a laxative [20, 21]. The phenolics and flavonoid phytochemicals of *C. fistula* have also been reported to be useful in treating skin diseases [14] . Therefore, in this study we are interested in the roles of *C. fistula* flower extract in cosmeceutical applications. The preventive effects of ECM degradation along with skin aging enzymes that include collagenase and MMP-2 activities, as well as tyrosinase, have been examined. Moreover, collagen and HA synthesis have been determined.

## Methods

### Chemicals and reagents

Dulbecco’s Modified Eagle Medium (DMEM), DQ gelatin and penicillin-streptomycin were supplied from Gibco (Grand Island, NY, USA). Fetal bovine serum (FBS) was supplied from Thermo Scientific (USA). Sirius Red/Fast Green Collagen Staining Kit was purchased from Chondrex, Inc. (Redmond, WA, USA). Sulforhodamine B reagent, hyaluronicacid and mushroom tyrosinase were obtained from Sigma-Aldrich (St. Louis, MO, USA).

### Plant materials

*C. fistula* flowers were obtained from Lampang Herb Conservation, Lampang Province, Thailand. A voucher specimen number (023197) was certified by Wannaree Charoensup, Botanist at the herbarium of the Flora of Thailand, Faculty of Pharmacy, Chiang Mai University, Thailand.

### Preparation of *Cassia fistula* flower extract

*C. fistula* flowers were dried in an airy room and then 500 g of dried flowers were finely ground into powder. After that, the powder was soaked in 50% ethanol and then shaken at 70 rpm for 24 h. After 24 h, the samples were then filtered through filter paper to separate the residue. The residue was soaked in 50% ethanol and shaken at 70 rpm for 24 h and this step was repeated 2 times. The filtrated samples were pooled together and evaporated by a rotary vacuum evaporator (BUCHI, Switzerland) to obtain the water fractions. Hexane was added to the water fraction at a ratio of hexane 2:1. The samples were shaken and allowed to separate over 30 min. The samples were separated into two fractions, which were hexane and water fractions. Then, the water fraction was collected and 10% charcoal was added for a 30 min period with mild stirring at room temperature. The samples were filtered through filter paper and celite to separate the charcoal. The samples were mixed with saturated butanol at a ratio of saturated butanol 2:1 and the samples were allowed to separate over a 12 h period and this step was repeated 3 times. The samples were separated into two fractions, which included water and butanol fractions. Water was further added to the butanol fractions to an equal volume and these fractions were allowed to evaporate through the use of a rotary vacuum evaporator. The evaporated samples were freeze-dried to obtain the *C. fistula* flower extract powder.

### Quantification of total phenolic content in *C. fistula* flower extract using UV-visible spectrophotometer

Total phenolic content in *C. fistula* flower extract was determined using the Folin-Ciocalteu assay. Briefly, 0.4 mL of *C. fistula* flower extract were mixed with 0.3 mL of 10% Folin-Ciocalteau reagent and incubated in a dark at room temperature for 3 min. Then, 0.3 mL of sodium carbonate solution was added and the solution was further incubated in the dark at room temperature for 30 min. The absorbance was evaluated at 765 nm using a UV-visible spectrophotometer. The concentration of the total phenolic content was calculated and compared with a standard curve for gallic acid (GA) at 0–20 μg/mL. The total phenolic content was reported as milligrams of GA equivalents per gram of *C. fistula.* flower extract (mg GAE/g extract).

### Quantification of phenolic compounds in *C. fistula* flower extract using HPLC analysis

Various types of phenolic compounds in *C. fistula* flower extracts were analyzed using HPLC analysis and were then compared with standard gallic acid, catechins, protocatecheuic acid, vanillic acid, chlorogenic acid, ferulic acid and coumaric acid. The flower extracts were dissolved in 50% ethanol and were further assessed by HPLC (Agilent Tecnologies, CA, USA) using reversed-phase C18 column (WATER, MA, USA). The mobile phase consisted of methanol (A) and 0.1% trifluoroacetic acid (TFA) in water (B) with gradient condition. The flow rate was set at 1.0 mL/min and the detection wavelength was recorded at 280 with a UV detector. The concentration levels of the phenolic compounds were calculated and compared with the standard curve considering the standard concentrations and peak areas (mg/g extract).

### Quantification of total flavonoid content in *C. fistula* flower extract using colorimetric assay

Total flavonoid content was determined by modified aluminium chloride (AlCl_3_) colorimetric assay as was previously described [22]. Briefly, 0.25 mL of flower extract were mixed with 0.125 mL of 5% sodium nitrite (NaNO_2_) and the solution was incubated for 5 min at room temperature. Then, 0.125 mL of 10% AlCl_3_ were mixed into the mixture and it was kept in the dark for 5 min. After which, 1 mL of sodium hydroxide (NaOH) was added and the solution was incubated for 15 min at room temperature in the dark. The absorbance was measured at 510 nm using a UV-visible spectrophotometer. The total flavonoid content was calculated and compared with the standard catechin values and expressed as mg catechin equivalent per gram of extract (mg CE/g extract).

### Cell cultures

Primary human skin fibroblasts were aseptically isolated from an abdominal scar after a surgical procedure involving a cesarean delivery at the surgical ward of CM Maharaj Hospital, Chiang Mai University (Chiang Mai, Thailand) (Study code: BIO-2558-03549 approved by Medical Research Ethics Committee, Chiang Mai University). Fat was removed from the starting material and it was soaked in DMEM containing anti-penicillin and streptomycin. After that, the skin was immersed in DMEM containing 1% protease (Dispase, Gibco, Grand Island, NY, USA) for 48 h at 4 °C. Epidermal layers were removed and the normal fibroblasts were isolated from the dermis. The fibroblasts were cultured in DMEM supplemented with 10% FBS, 2 mM L-glutamine, 50 U/mL penicillin, and 50 μg/mL streptomycin. The cells were maintained in a 5% CO_2_ humidified incubator at 37 °C.

### Cell viability assay

The cell viability assay of *C. fistula* flower extract against skin fibroblast cells was measured using a sulforhodamine B (SRB) assay as was previously described [23]. Briefly, the cells (8x10^3^cells/well) were seeded in a 96-well plate and incubated at 37 °C, 5% CO_2_ for 24 h. After that, the cells were treated with or without various concentrations (0–200 μg/mL) of *C. fistula* flower extract for 48 h. After 48 h, 10% (w/v) Trichloroacetic acid (TCA) was added to the cells and the cells were then incubated at 4 °C for 1 h. The medium was removed and the cells were rinsed with slow running tap water. 0.057% (w/v) SRB solution (100 μL) was added to each well and the cells were incubated for 30 min at room temperature. The SRB solution was removed and then the cells were washed 4 times with 1% (v/v) acetic acid and they were allowed to dry at room temperature. The dye was dissolved with 10 mM Tris base solution (pH 10.5) and the absorbance was measured at 510 nm using a microplate reader.

### Collagen synthesis assay

Total intracellular collagen in the fibroblast cells was determined using Sirius Red/Fast Green Collagen Staining Kit (Chondrex, Inc, Redmond, WA, USA) according to the manufacturer’s instructions. Briefly, skin fibroblast cells were seeded in 24 well plates for 24 h at 37 °C, 5% CO_2_. After that, the cells were pre-treated with the serum free DMEM medium for 24 h. The medium was removed and the cells were then incubated with or without *C. fistula* flower extract (0-150 μg/mL) for 48 h. After 48 h, the cultured medium was removed. Then, the cells were washed with PBS and fixed with Kahle fixative for 10 min. The fixative was removed and the cells were washed with PBS. The Sirius Red/Fast Green dye solution was added to each well and the specimens were incubated for 30 min at room temperature. The dye was removed and the cells were washed four times with DI water or until the fluid was colorless. Finally, the extraction buffer was added and the absorbance was measured at 540 nm and 605 nm using a UV-visible spectrophotometer.

### Collagenase activity assay

The collagenase activity was measured using modified fluorogenic DQ™-gelatin assay as has been previously described [24]. Briefly, various concentrations of *C. fistula* flower extract (0–200 μg/mL) were added in 96 well plates. One U/ml of collagenase was added in each well (100 μL/well). After that, 15 μg/mL of gelatin (DQ gelatin) was added and the mixtures were incubated for 10 min. The rate of proteolysis was determined by measuring the absorbance at 2 min intervals for 20 min using a microplate reader at an excitation wavelength of 485 nm and an emission wavelength of 528 nm. Enzyme activity was estimated by examining the slope of linear regression between time and absorbance over a 2-6 min period.

### MMP-2 activity assay

The MMP-2 activity was determined by gelatin zymography as was previously described [25]. Fibroblast cells were cultured in DMEM serum-free medium for 24 h. After that, the culture supernatant was collected. The culture supernatant was subjected to 10% polyacrylamide gels containing 0.1% w/v of gelatin under non-reducing conditions. The gels were washed twice with 2.5% v/v of Triton X-100 for 30 min at room temperature to remove SDS. The gel slab was cut into slices which corresponded to the lanes and then the slices were put into different tanks and were incubated with an activating buffer (50 mM Tris-HCl, 200 mM NaCl, 10 mM CaCl_2_, pH 7.4) containing various concentrations of *C. fistula* flower extract (0–200 μg/mL) at 37 °C for 18 h. After that, a strip of the gels was washed and stained with Coomassie Brillant Blue R (0.1% w/v) and then was destained in 30% methanol and 10% acetic acid. MMP-2 activity appeared as a clear band against a blue background. Digestion bands were quantitated by the Image J program.

### Hyaluronic Acid (HA) synthesis assay by ELISA

The effect of *C. fistula* flower extract on HA synthesis was determined using an ELISA kit as previously described [26]. The fibroblast cells (5.0 × 10^4^ cells/well) were seeded in 24-well-plates for 24 h at 37 °C, 5%CO_2_. The cells were pre-treated in serum free medium for 6 h and were then treated with or without various concentrations of *C. fistula* flower extract (0–200 μg/mL) for 48 h. The cultured medium was collected and HA synthesis was measured by ELISA. The absorbance was measured at 450 and 570 nm using a microplate reader. The HA concentration in the cultured supernatant acquired from the treated fibroblast cells was calculated and compared with the standard curve of HA.

### Tyrosinase inhibition assay

Tyrosinase inhibition assay was determined as was previously described [27]. Various concentrations of *C. fistula* flower extract were added to a 96-well-plate. The reaction was carried out in a sodium buffer (pH 6.4) containing 100 U/mL mushroom tyrosinase. L-DOPA substrate (1 mM) was added into the reaction mixture and it was then incubated for 10 min at room temperature. The change of the absorbance at 490 nm was measured every 1.5 min for 15 min using a microplate reader. The percent inhibition of tyrosinase was calculated by the following formula:

$$ \mathrm{Tyrosinase}\ \mathrm{inhibition}\ \left(\%\right) = \left[\left(\mathrm{A}-\mathrm{B}\right)/\mathrm{A}\right] \times 100, $$

A = slope of control at 490 nm

B = slope of test at 490 nm

### Antioxidant activity assay

The antioxidant activity of the *C. fistula* flower extract was determined using 1, 1-diphenyl-2-picrylhydrazyl (DPPH)-free radical activity, as previously described [28]. Various concentrations of the flower extract (0–100 μg/mL) were prepared in methanol. 1 mL of 0.002% of DPPH was added to 1 mL of the flower extract solution and the mixture was kept in the dark for 30 min. Absorbance was measured at 517 nm using a spectrophotometer. The % inhibition was calculated and compared with standard vitamin E (1–100 μg/mL) using the following formula:

$$ \mathrm{Percent}\ \mathrm{inhibition}\ \mathrm{of}\ \mathrm{DPPH}\ \mathrm{activity} = \frac{{\mathrm{A}}_{\mathrm{control}\ \mathrm{at}\ 517\ \mathrm{nm}}-{\mathrm{A}}_{\mathrm{sample}\ \mathrm{at}\ 517\ \mathrm{nm}}}{{\mathrm{A}}_{\mathrm{control}\ \mathrm{at}\ 517\ \mathrm{nm}}}\times 100 $$

The antioxidant activity of the *C. fistula* flower extracts was confirmed using ABTS assay, as was previously described [29]. ABTS [2, 2′- azino-bis (ethylbenzthiazoline-6-sulfonic acid)] radical cation was prepared by mixing 7 mM ABTS stock solution with 2.45 mM potassium persulfate (K_2_S_2_O_8_) (1/1, v/v). The mixture was incubated in the dark for 12–16 h until the reaction was completed. The assay was conducted on 990 μL of ABTS solution and 10 μL of the flower extract (0–4 μg/mL). After 6 min, the absorbance was recorded immediately at 734 nm using a spectrophotometer. The percent inhibition of ABTS activity of the flower extract was calculated using the following equation:

$$ \mathrm{Percent}\ \mathrm{inhibition}\ \mathrm{of}\ \mathrm{ABTS}\ \mathrm{activity} = \kern0.5em \frac{{\mathrm{A}}_{\mathrm{control}\ \mathrm{at}\ 734\ \mathrm{nm}}-{\mathrm{A}}_{\mathrm{sample}\ \mathrm{at}\ 734\ \mathrm{nm}}}{{\mathrm{A}}_{\mathrm{control}\ \mathrm{at}\ 734\ \mathrm{nm}}}\times 100 $$

### Statistical analysis

All data are presented as mean ± standard deviation (S.D.) values. Statistical analysis was analyzed by Prism version 6.0 software using one-way ANOVA with Dunnett’s test. Statistical significance was determined at * *p* < 0.05, ** *p* < 0.01, ***p < 0.001 or **** *p* < 0.0001.

## Results

### Phytochemical characterization of *C. fistula* flower extract

**Fig. 1:**
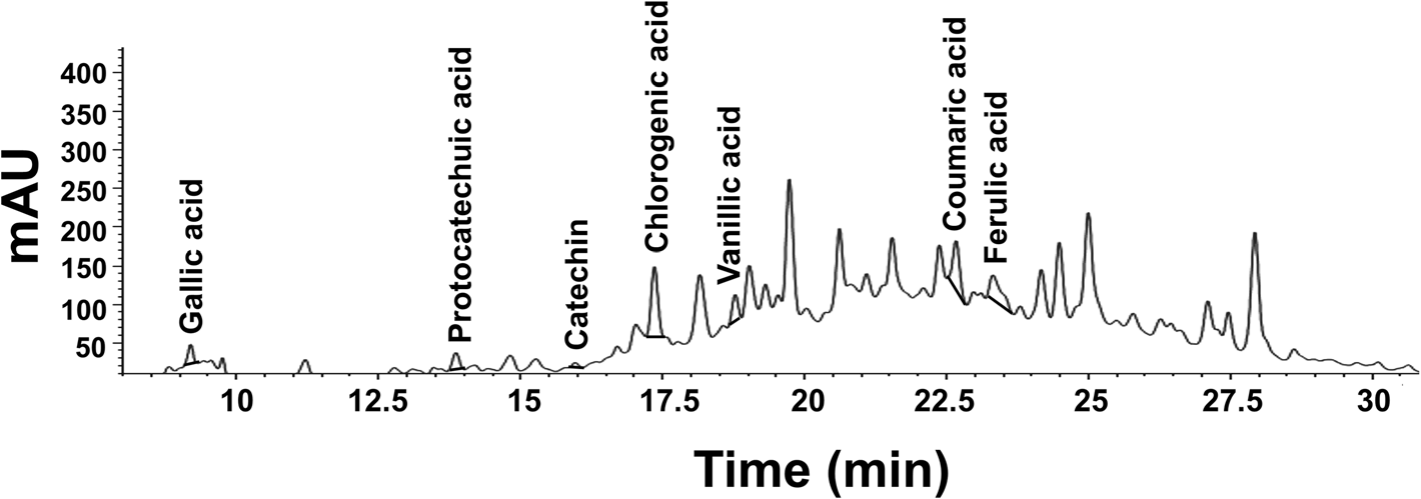
HPLC fingerprint of *C. fistula* flower extract. The HPLC fingerprint of the flower extract was evaluated using reversed-phase C18 column and its mobile phase consisted of methanol and 0.1% trifluoroacetic acid (TFA). The flow rate was set at 1.0 mL/min and the detection wavelength was 280 and 325 nm

After the step involving freeze-drying the specimens, the *C. fistula* flower extract was weighed and further subjected to phytochemical and biological studies. 18.08 g of *C. fistula* flower extract was obtained from 500 g of raw material and the percent yield of the flower extract was 3.62. The HPLC fingerprint of the *C. fistula* flower extracts was determined by HPLC analysis (Fig. 1). HPLC profile that showed the peak of the phenolic compounds was labeled in terms of the individual components at each retention time. The characterization of the phenolic compounds of *C. fistula* flower extract is shown in Table 1. The total phenolic content in the flower extract was 275.32 ± 14.21 mg GAE/g extract. The hydroxybenzoic acid derivatives, including vanillic acid and protocatechuic acid, were found to be 0.95 ± 0.09 and 2.56 ± 0.42 mg/g extract, respectively, whereas gallic acid was recorded at 0.60 ± 0.02 mg/g extract. The hydroxycinnamic acid derivatives, including coumaric acid, ferulic acid, and chlorogenic acid, were detected at 0.39 ± 0.08, 0.80 ± 0.09, and 0.83 ± 0.03 mg/g extract, respectively. The flavonoid content was 27.62 ± 3.56 mg CE/g extract. Catechin, as a flavonol derivative, was measured at 1.10 ± 0.00 mg/ g extract.

**Table 1:** The phenolic compound content in *C. fistula* flower extracts

Compounds (mg/g extract)	*C. fistula* flower extracts
Total phenolic content	275.32 ± 14.21
Vanillic acid	0.95 ± 0.09
Protocatechuic acid	2.56 ± 0.42
Gallic acid	0.60 ± 0.02
Coumaric acid	0.39 ± 0.08
Ferulic acid	0.80 ± 0.09
Chlorogenic acid	0.83 ± 0.03
Total flavonoid content	27.62 ± 3.56
Catechins	1.10 ± 0.00

### Effect of *C. fistula* flower extract on fibroblast cells cytotoxicity

**Fig. 2:**
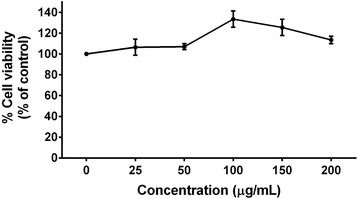
Cytotoxicity of *C. fistula* flower extract on skin fibroblasts. The cells were treated with or without various concentrations of the flower extract (0–200 μg/mL) for 48 h. The cell viability was determined using SRB assay. All assays have been performed in triplicate and the mean ± standard deviations are shown

Cytotoxicity testing of *C. fistula* flower extract on fibroblast cells was measured using SRB assay. Fibroblast cells were treated with or without various concentrations of the flower extract (0–200 μg/mL). After a treatment of 48 h, the flower extract was found to have no effect on skin fibroblast cell growth (0–200 μg/mL). The IC_20_ and IC_50_ of the flower extract was found to be more than 200 μg/mL, which could also be applied in other experiments without toxicity (Fig. 2).

### Effect of *C. fistula* flower extract on collagen synthesis in human skin fibroblast cells

**Fig. 3:**
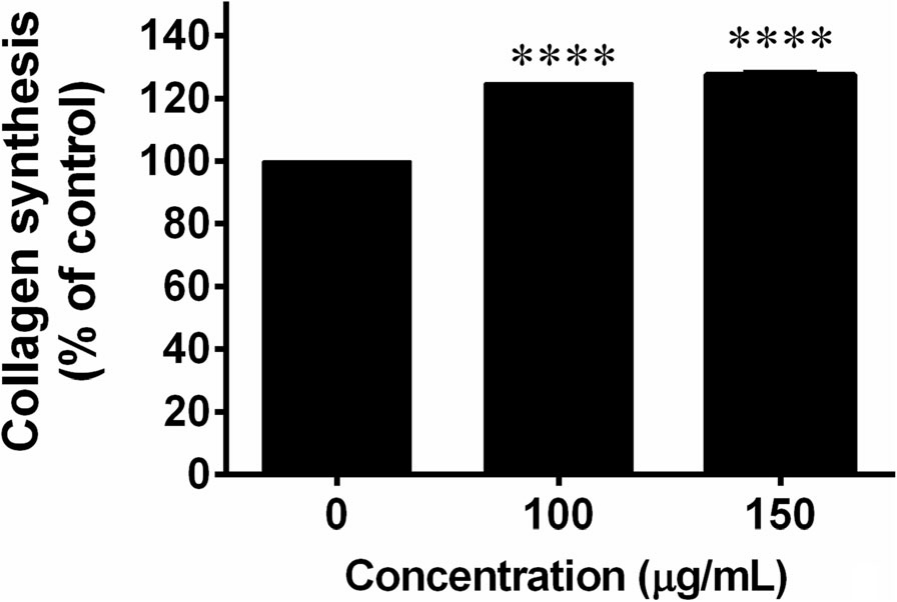
The effects of *C. fistula* flower extract on collagen synthesis were determined using a collagen kit. Fibroblast cells were pre-treated in serum free medium for 24 h. and were further treated with or without various concentrations of flower extract (0–150 μg/mL) for 48 h. The cells were stained with Sirius Red/Fast Green dye and the absorbance was determined using a UV-spectrophotometer. All assays have been performed in triplicate and the mean ± standard deviations are shown as ****p < 0.0001 versus the non-treated cells

Collagen plays a key role in both skin wound healing and the skin rejuvenation process. Collagen synthesis from skin fibroblast cells was achieved using a Sirius Red/Fast Green Collagen Staining Kit. Collagen synthesis from fibroblast cells was found to have significantly increased in a dose dependent manner after cells were treated with various concentrations (100–150 μg/mL) of the flower extract (Fig. 3).

### Effect of *C. fistula* flower extract on collagenase activity

**Fig. 4:**
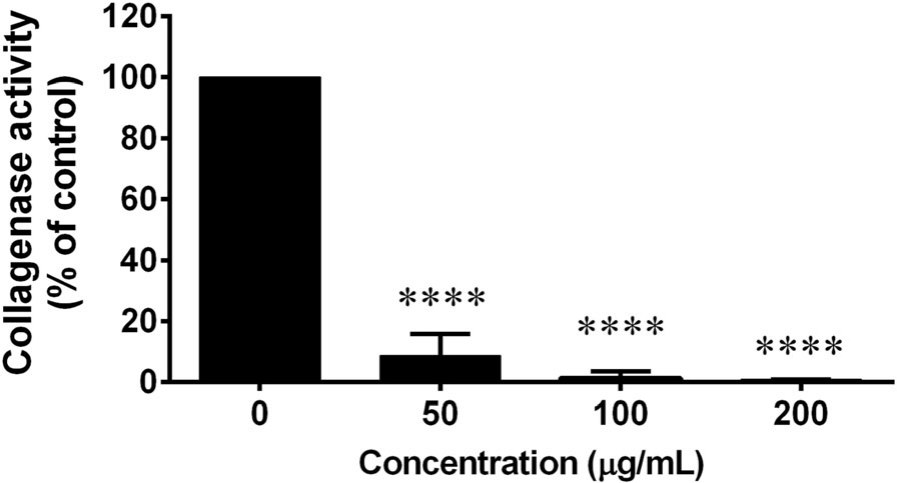
The effect of *C. fistula* flower extract on collagenase activity was determined using fluorogenic DQ™-gelatin assay. Various concentrations of the flower extract (0–200 μg/mL) were mixed with collagenase. After that, DQ gelatin substrate was added and incubated for 10 min. The rate of proteolysis was evaluated using a microplate reader. All assays have been demonstrated in triplicate and the mean ± standard deviations are shown as ****p < 0.0001 versus the control without the extract

Collagenases are the enzymes that digest native collagen in the triple helix region. Therefore, the inhibition of collagenase activity could protect against collagen breakdown. Collagenase activity was measured using fluorogenic DQ™-gelatin assay. Collagenase activity was dramatically decreased in a dose dependent manner after treating the fibroblasts with the flower extract. At high concentrations, the flower extract (200 μg/mL) could completely inhibit collagenase activity (Fig. 4).

### Effect of *C. fistula* flower extract on MMP-2 activity

**Fig. 5:**
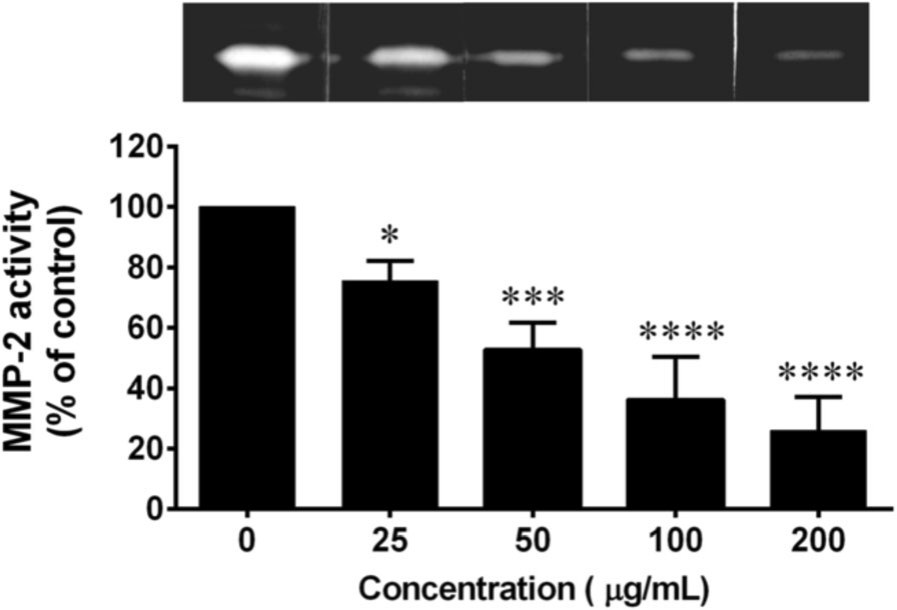
The effects of *C. fistula* flower extract on MMP-2 activity were determined using gelatin zymography. Fibroblast cells were cultured in DMEM serum-free medium for 24 h and the culture supernatant was subjected to 10% polyacrylamide gels containing 0.1% w/v of gelatin under non-reducing conditions. The gels were incubated with or without various concentrations of red rice extract (0–200 μg/mL) and were then stained with Coomassie Brillant Blue R. The digestion bands were quantitated by the Image J program. All assays have been performed in triplicate and the mean ± standard deviations are shown as *p < 0.05, ***p < 0.001 and ****p < 0.0001 versus the non-treated culture supernatant

MMP-2 is an enzyme that is involved in the breakdown of the extracellular matrix (ECM) and plays an important role in influencing normal homeostasis, aging and the wound-healing of the skin. MMP-2 activity was measured using gelatin zymography. It was determined that MMP-2 secreted from skin fibroblast cells could digest gelatin in the gel. However, after the gel was incubated with various concentrations of the flower extract (50–200 μg/mL), the level of MMP-2 activity was significantly reduced in a dose dependent manner (Fig. 5).

### Effect of *C. fistula* flower extract on HA synthesis in human skin fibroblast cells

**Fig. 6:**
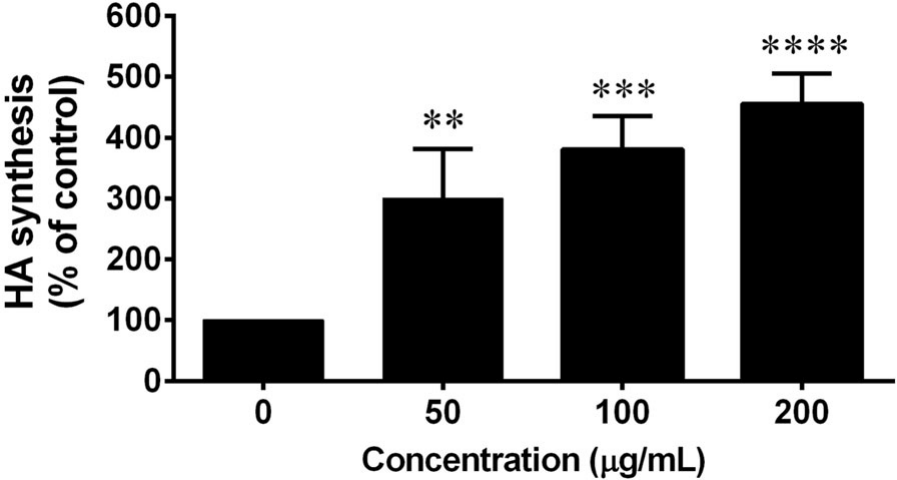
The effects of *C. fistula* flower extract on HA synthesis were determined using an ELISA kit. Fibroblast cells were pre-treated in serum free medium for 6 h and were further treated with or without various concentrations of the flower extract (0–200 μg/mL) for 48 h. The cultured medium was collected for ELISA and the absorbance was measured at 450 and 570 nm using a microplate reader. All assays have been performed in triplicate and the mean ± standard deviations are shown as **p < 0.01, ***p < 0.001 and ****p < 0.0001 versus the non-treated cells

HA synthesis from skin fibroblast cells was evaluated using an ELISA kit. After the cells were treated with various concentrations of the flower extract for 48 h, HA synthesis was found to have significantly increased in a dose dependent manner (50–200 μg/mL). After the fibroblasts were treated with flower extracts at 200 μg/mL, HA synthesis was induced at a level that was four-fold when compared with the non-treated cells (Fig. 6).

### Effect of *C. fistula* flower extract on tyrosinase activity

**Fig. 7:**
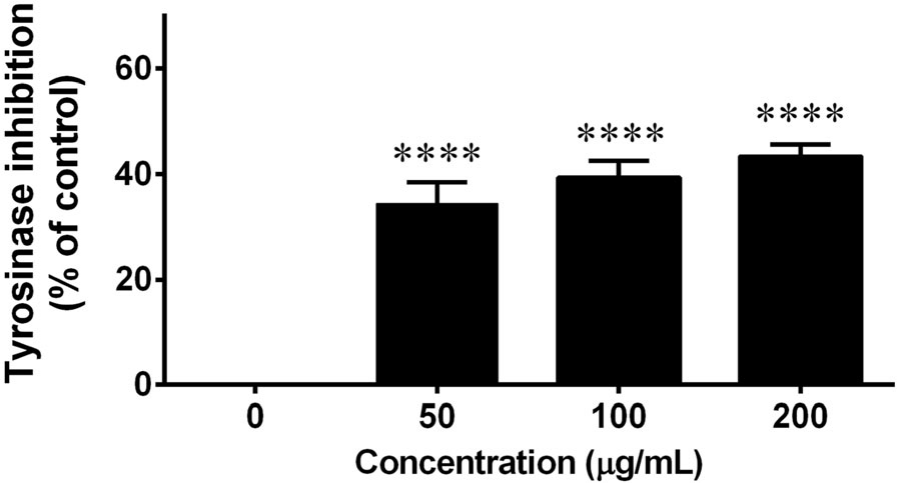
Tyrosinase inhibition assay was determined in the *C. fistula* flower extract. Various concentrations of the flower extract were mixed with 100 U/mL of mushroom tyrosinase in a sodium buffer (pH 6.4). L-DOPA substrate (1 mM) was added into the reaction mixture and it was then incubated for 10 min. The change of the absorbance was measured at 490 using a microplate reader. All assays have been demonstrated in triplicate and the mean ± standard deviations are shown ****p < 0.0001 versus the control without the extract

Tyrosinase is an enzyme that is involved in the rate-limiting step for the control of melanin production. Therefore, the inhibition of tyrosinase activity tends to induce skin whitening due to a reduction of melanin synthesis. When the tyrosinase enzyme was incubated with the flower extract, it could inhibit tyrosinase activity in a dose dependent manner at a concentration of 50–200 μg/mL (Fig. 7).

### Anti-oxidant activity of *C. fistula* flower extract

**Fig. 8:**
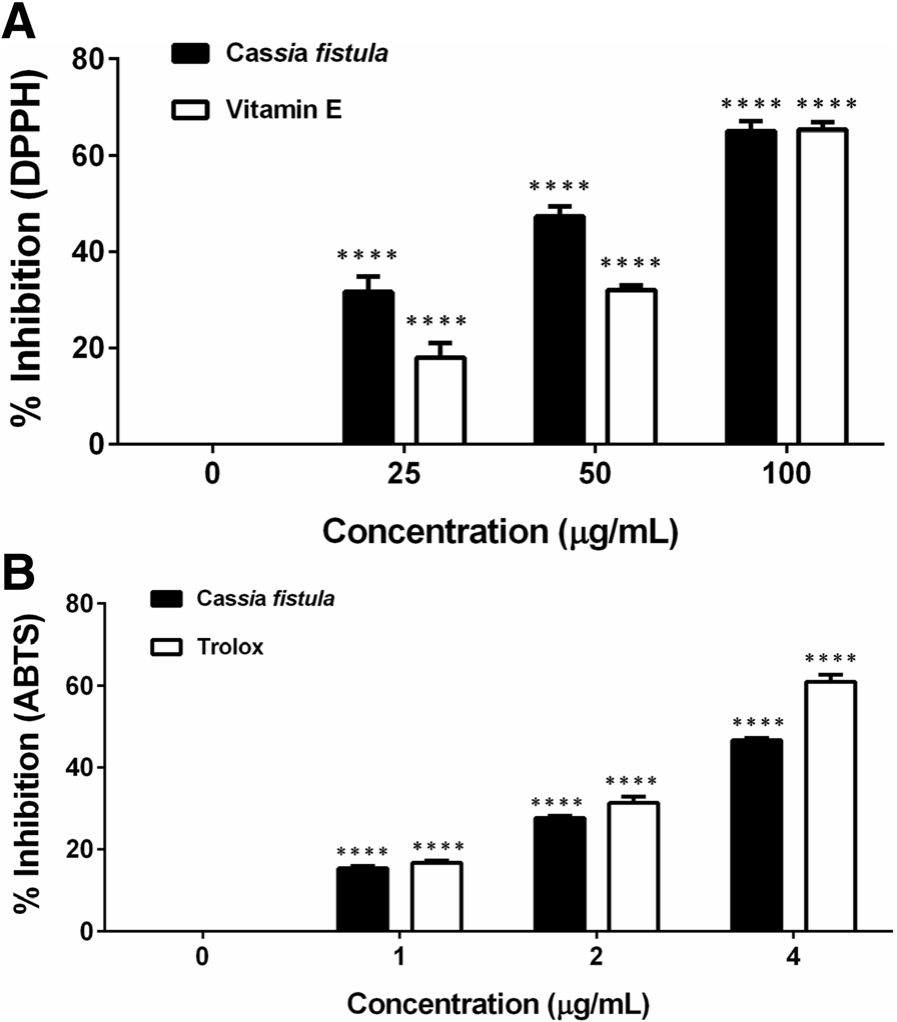
Free radical scavenging activity of *C. fistula* flower extracts was determined using DPPH assay (**a**) and ABTS assay (**b**). 1 mL of 0.002% of DPPH was added to 1 mL of the flower extract solution and it was kept in the dark for 30 min. Absorbance was measured at 517 nm using a spectrophotometer. Percent inhibition of the DPPH radical was calculated and compared with that of standard vitamin E. For ABTS assay, the flower extract was mixed with ABTS solution and was then incubated for 6 min. Absorbance was measured at 734 nm using a spectrophotometer. Percent inhibition of the ABTS radical was calculated and compared with that of the standard trolox. All assays have been demonstrated in triplicate and the mean ± standard deviations are shown as ****p < 0.0001 versus control

The free radical scavenging activity of *C. fistula* flower extract was then examined by DPPH and ABTS assay. *C. fistula* flower extract dose dependently inhibited oxidant activity. Vitamin e and trolox were used as a positive control in each experiment. *C. fistula* flower extract exhibited scavenging activity (DPPH assay) at a value of 65% at 100 μg/mL. At 25 μg/mL of the flower extract, the radical scavenging activity was found to still be approximately 33% and the IC_50_ of the flower extract and vitamin E were recorded at 70 and 72 μg/mL, respectively. These findings indicate that *C. fistula* flower extract is a potent antioxidant and in this capacity is comparable to vitamin E (Fig. 8a). To confirm the anti-oxidant activity of the flower extract, the ABTS assay was determined and showed % inhibition of 47% at 4 μg/mL and IC_50_ of the flower extract and the trolox were 4.8 and 3 μg/mL, respectively, which was in accordance with the data acquired from the DPPH assay (Fig. 8b).

## Discussion

Extrinsic and/or environmental factors cause skin aging signs which can include wrinkles and pigment spot formations [30]. In previous studies, UV-radiation that is known to induce skin aging has been a major topic of research through the focus of the pathogenesis and molecular mechanisms. The generation of ROS can stimulate skin inflammation leading to the activation of transcription factors which regulate the degradation of the skin collagen and the extracellular matrix (ECM) [30]. These events result in a loss of the skin’s ability to resist stretching, which ultimately leads to skin aging.

*C. fistula* flower extract has been traditionally used for the treatment of skin diseases, abdominal pain and wound-healing [17]. Our results show that the major phytochemicals represented in the *C. fistula* flower extract were the phenolic compounds and flavonoids. The main phenolic components in the *C. fistula* flower extract were protocatecheuic acid followed by vanillic acid, chlorogenic acid and ferulic acid. In addition, Bahorun T, et al have reported that *C.fistula* flowers contain various types of flavonoids including kaempferol, rhein, fistulin, alkaloids and triterpenes. Among those phytochemical compounds, kamempferol, catechins, ferulic acid, chlorogenic acid and protocatecheuic acid have been proven to exhibit antiaging activities. In this study, the antiaging activity of the *C. fistula* flower extract was investigated in order to determine the effects of the extract on collagen, HA and melanin production. Our results indicate that high concentrations of *C. fistula* flower extract (200 μg/ml) did not have an effect on the viability of human skin fibroblast cells. Therefore, *C. fistula* flower extracts could be safe in applications to the human skin.

Collagen synthesis in skin fibroblasts plays a major role in skin rejuvenation. The reduction of types I and III procollagen synthesis is a critical feature of aged skin leading to skin thinning and the increased fragility of skin. [31]. Hence, the inhibition of collagen synthesis or a loss in the function of collagen results in chronologically aged skin. Our results indicate that the *C. fistula* flower extract significantly induced collagen synthesis from the skin fibroblasts and also dramatically inhibited collagenase activity, which is the enzyme involved in collagen breakdown. Chronologically aged skin induced by UV radiation also occurs through an increase in MMPs production, including, MMP-1, MMP-2, MMP-3 and MMP-9, which causes an imbalance of collagen synthesis by the induction of collagen or by ECM degradation [32]. This is the first report indicating that *C. fistula* flower extracts significantly inhibit MMP-2 activity in a dose dependent manner. For applications in cosmetic formulations, the *C. fistula* extract at a concentration of 50 μg/mL should be considered. These findings suggest that *C. fistula* flower extract possesses useful booster collagen benefiting the skin via reduced collagen breakdown.

Glycosaminoglycans (GAGs) or hyaluronic acid (HA), a major component of extracellular matrix, is induced during wound-healing and skin regeneration and keeps skin hydrated [33]. Environmental factors such as UV radiation induce the type of skin aging that results in a loss of skin elasticity causing skin to become wrinkled by decreasing HA synthesis [34]. This result indicates that the *C. fistula* flower extract dramatically increased HA synthesis in a dose dependent manner. Hence, the flower extract can enhance skin moisture and can result in skin being less dry by increasing HA synthesis.

Hyperpigmentation causes human skin aging and occurs as a result of both internal and external factors including those related to hormones, UV exposure, drugs, and the presence of various chemicals [4]. Melanin biosynthesis is a pathway that appears in melanocytes. Hyperpigmentation is particularly obvious in darker skin and is often difficult to treat. Cosmetic scientists have conducted various *in vivo* and *in vitro* studies on skin lightening agents. The key enzyme that regulates melanin synthesis is tyrosinase, which is involved in two steps of melanin synthesis, including the hydroxylation of tyrosine to β-3,4-dihydroxyphenylalanine (DOPA) and the oxidation of DOPA to DOPA quinone [4]. Our results indicate that the *C. fistula* flower extract can successfully reduce tyrosinase activity. This result was similar to that of certain previous studies, which showed that *C. fistula* pods have displayed skin whitening activity *in vitro* and *in vivo* by using tyrosinase activity as an endpoint bioassay [35]. Therefore, it can be concluded that this *C. fistula* flower extract can reduce hyperpigmentation in human skin. Previous studies have shown that some parts of the *C. fistula* plant exhibited anti-oxidant activity [36–38]. The aqueous and methanolic extracts of the *C. fistula* bark showed the free radical scavenging effect of DPPH in a dose dependent manner [38]. The hydroalcoholic extract of the *C. fistula* flower and fruit pulp showed antioxidant activity by inhibiting DPPH and hydroxyl radicals [36, 37]. Additionally, our study on the anti-oxidant activity of the butanolic extract of the *C. fistula* flower similarly displayed the free radical scavenging effect of DPPH and ABTS in a dose dependent manner.

## Conclusions

These results indicate that *C. fistula* flower extract displays a high potential for anti-aging in normal skin fibroblast cells, both in terms of the inhibition of wrinkles and a decrease in the number of pigment spots. *C. fistula* flower extract could prevent skin aging via an increase in collagen and HA production. Moreover, the flower extract also inhibited collagenase, MMP-2 and tyrosinase activity, all of which are involved in skin aging. Therefore, *C. fistula* extract that has displayed a non-toxic effect might be an alternative ingredient for use in cosmetics or supplements that are being developed for anti-aging applications.

## Abbreviations

ABTS: Azino-bis (ethylbenzthiazoline-6-sulfonic acid)
DI water: Deionized water
DMEM: Dulbecco’s Modified Eagle Medium
DPPH: Diphenyl-2-picrylhydrazyl
ECM: Extracellular matrix
ERK: Extracellular-signal-regulated kinases
FBS: Fetal bovine serum
g: Gram
GA: Gallic acid
h: Hour
HA: Hyaluronic acid
HPLC: High-performance liquid chromatography
JNK: c-Jun N-terminal protein kinase
mg: Milligram
min: Minute
mL: Milliliter
mM: Millimolar
MMP-2: Matrixmelloproteinase-2
MMPs: Metalloproteinases
nm: Nanometer
PBS: Phosphate buffer saline
rpm: Round per minute
SDS: Sodium dodecyl sulfate
SRB: Sulforhodamine B
TCA: Trichloroacetic acid
TFA: Trifluoroacetic acid
μg: Microgram
μL: Microliter
